# Supplementary material for: Integrating high resolution drone imagery and forest inventory to distinguish canopy and understory trees and quantify their contributions to forest structure and dynamics
Source: PLoS One. 2020 Dec 10;15(12):e0243079. doi: 10.1371/journal.pone.0243079 (PMC7728260; doi:10.1371/journal.pone.0243079)
Supplement: S1 Table — (DOCX) [file pone.0243079.s001.docx]

| SIZE CLASS | N CANOPY | P CANOPY |
| --- | --- | --- |
| 10-20 | 158 | 0.21 |
| 20-30 | 152 | 0.57 |
| 30-40 | 108 | 0.86 |
| 40-50 | 46 | 0.87 |
| 50-60 | 19 | 0.90 |
| 60-70 | 6 | 0.86 |
| 70-80 | 7 | 1.00 |
| 80-90 | 1 | 1.00 |
| 90-100 | 0 | 0.00 |
| 100-110 | 0 | 0.00 |
| 110-120 | 1 | 1.00 |
