# Supplementary figures and images for: Integrating high resolution drone imagery and forest inventory to distinguish canopy and understory trees and quantify their contributions to forest structure and dynamics
Source: PLoS One. 2020 Dec 10;15(12):e0243079. doi: 10.1371/journal.pone.0243079 (PMC7728260; doi:10.1371/journal.pone.0243079)

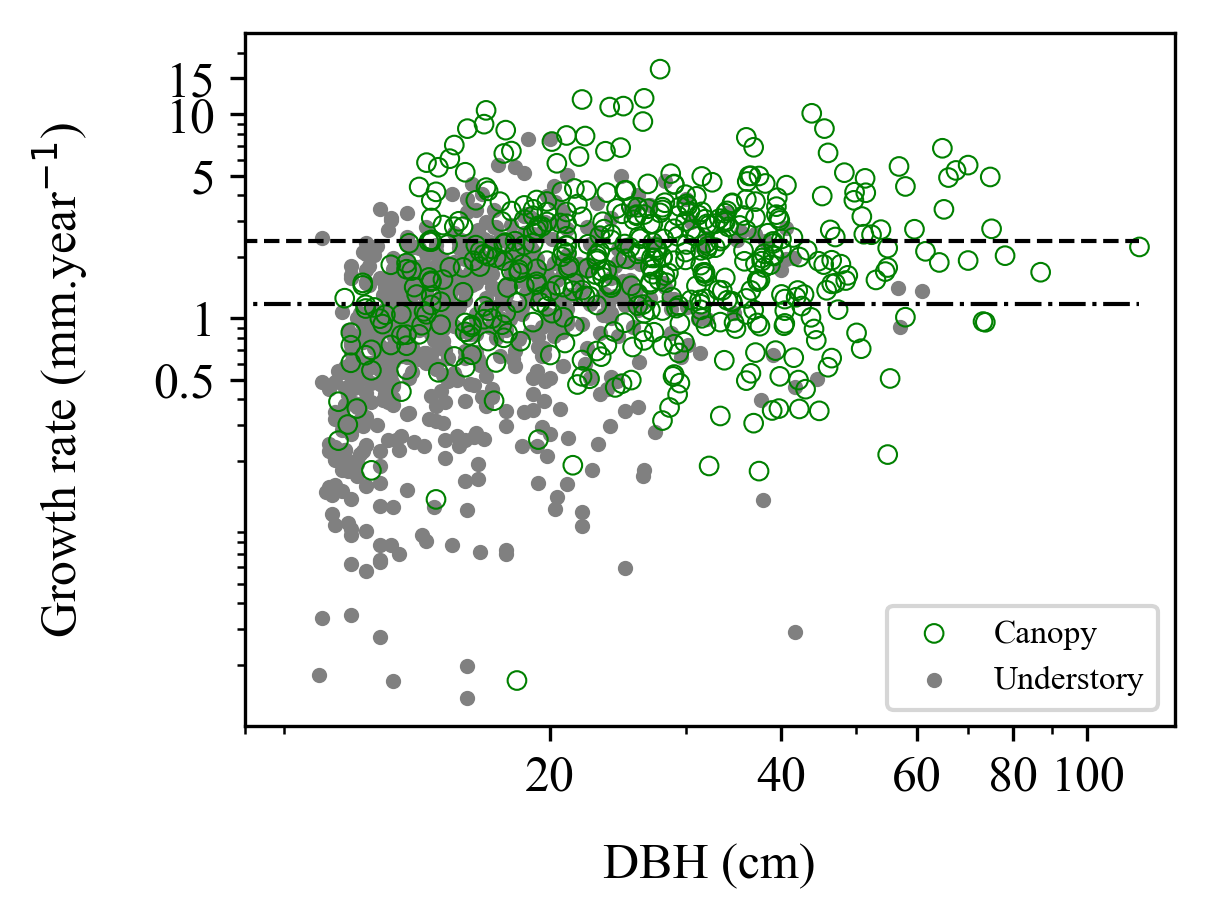

Supplement: S1 Fig — The dashed and dash-dot lines show the mean growth rates of 2.34 and 1.18 mm.year-1 for the canopy and understory groups, respectively. (TIF) [file pone.0243079.s002.tif]

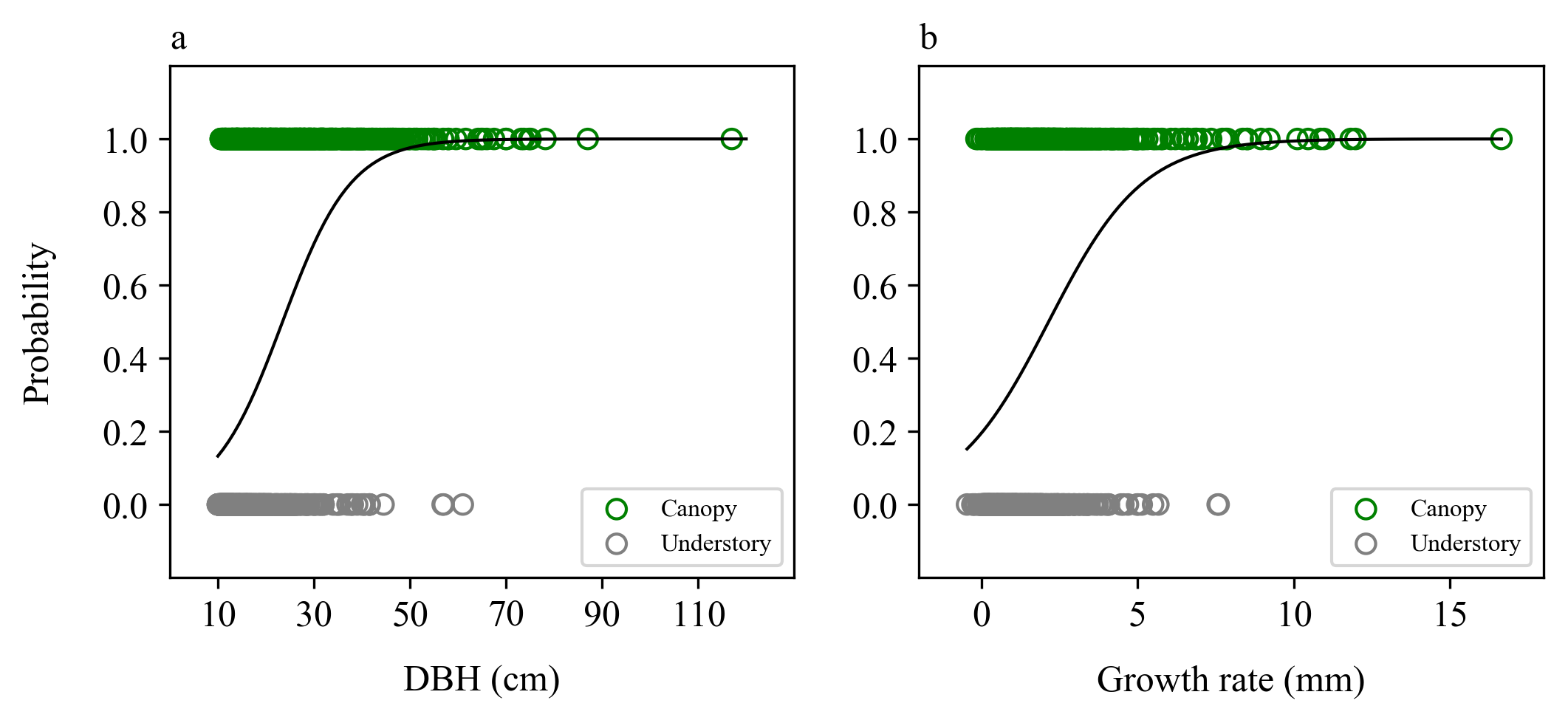

Supplement: S2 Fig — Canopy status in relation to DBH (a) and MAI (b) for individual trees (points), together with fitted logistic regressions (lines). The fitted lines cross 50% for at DBH of 23.5 cm and MAI of 2.2 mm.year-1. (TIF) [file pone.0243079.s003.tif]

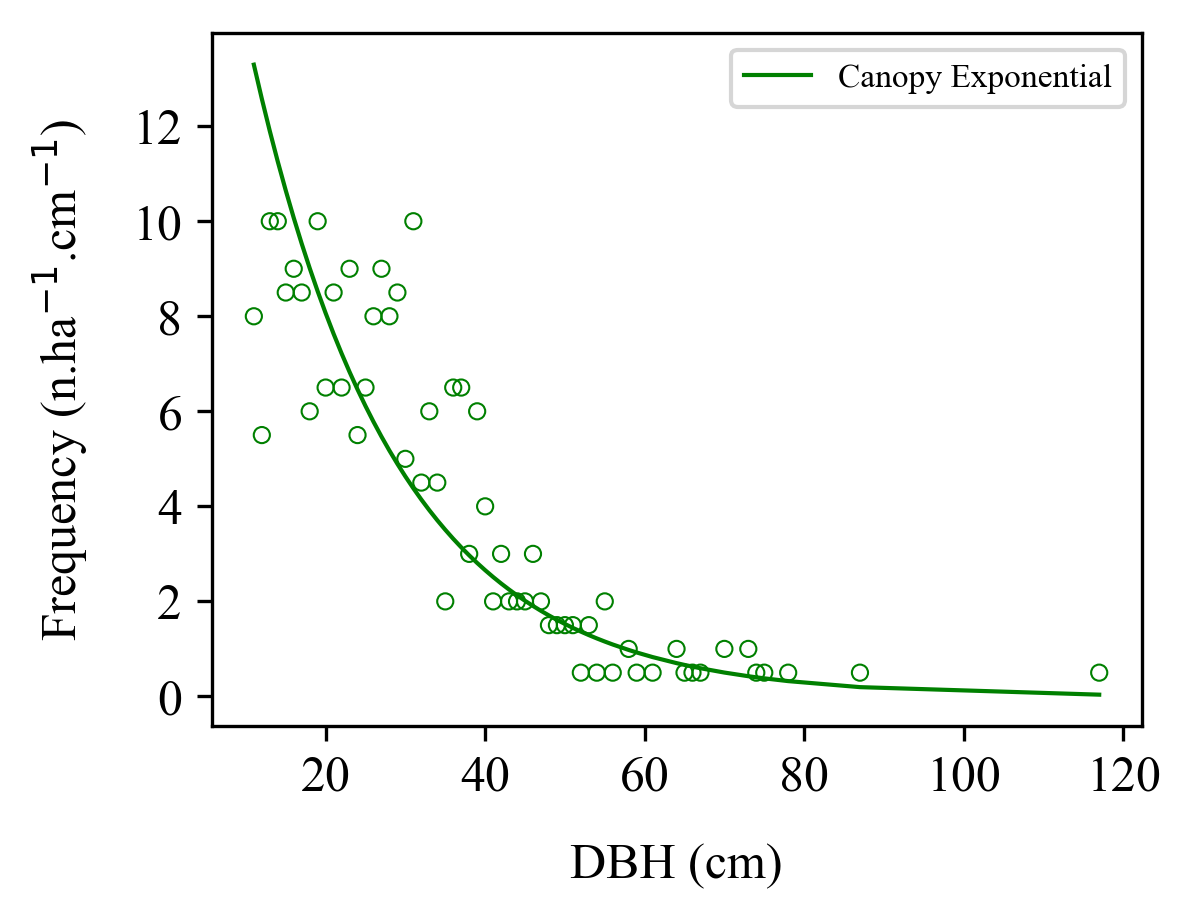

Supplement: S3 Fig — Observed size distribution of canopy trees (green circles) together with the exponential fit probability density functions (green line). The x-axis is the stem diameter class in cm, on a linear scale; the y-axis is the frequency of individuals per hectare per 1-cm size class. (TIF) [file pone.0243079.s004.tif]

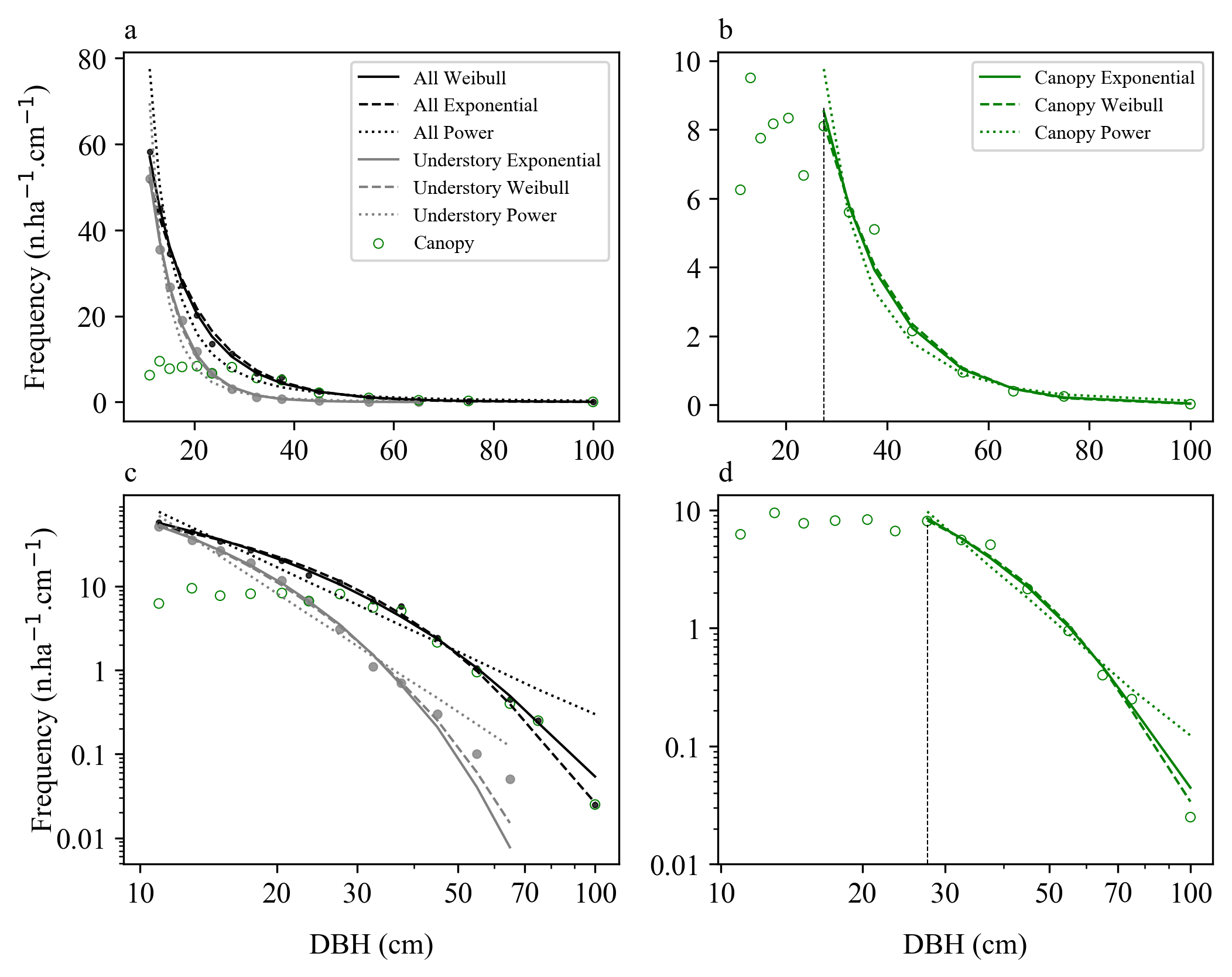

Supplement: S4 Fig — All 1244 trees with DBH > 10 cm (black points), understory trees (gray points), and canopy trees (green circles), shown together with fit probability density functions (lines). Distributions are shown on linear (top) and log (bottom) scales. The vertical dashed black line indicates the minimum diameter (25 cm) for inclusion in the canopy tree fits. The Weibull distribution (Eq 6) had the best fit for all individuals combined; the exponential distribution (Eq 4) had the best fit for understory trees as well as for canopy trees with DBH ≥ 25 cm (solid lines). The other fits are shown by dashed and dotted lines. Whereas data are graphed here for 10-cm size classes for visualization purposes, fits were carried out using 1-cm size classes (parameter values in Table 2). (TIF) [file pone.0243079.s005.tif]

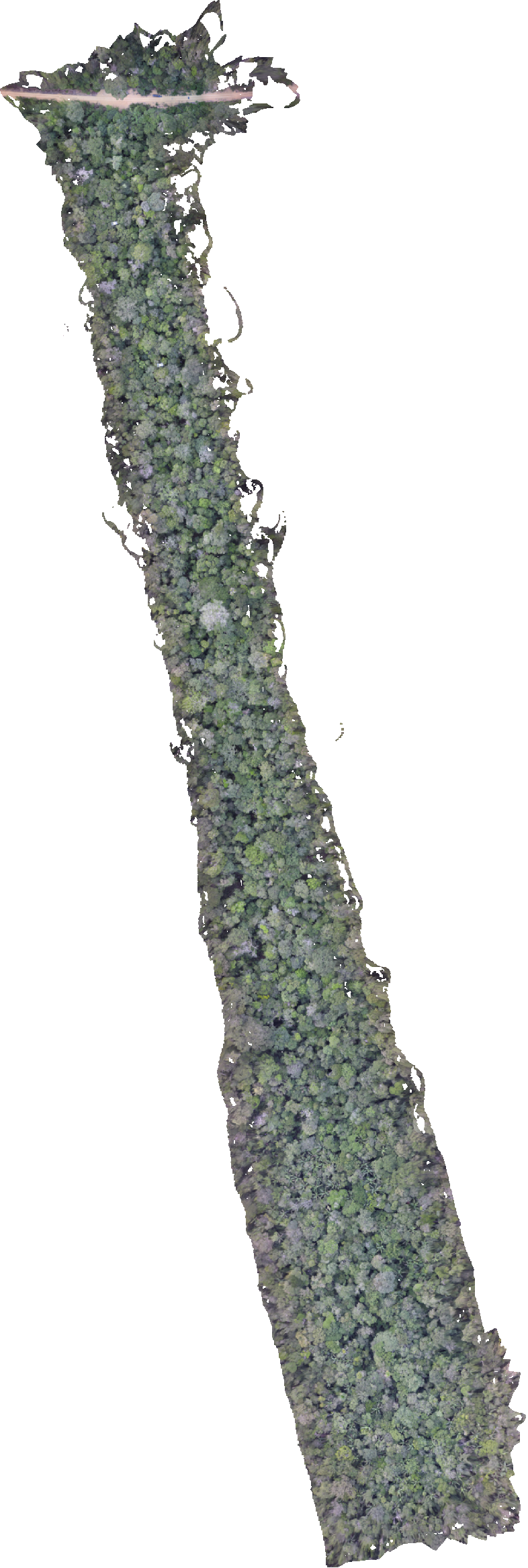

Supplement: S4 File — (TIF) [file pone.0243079.s009.tif]
