# Supplementary material for: Integrating high resolution drone imagery and forest inventory to distinguish canopy and understory trees and quantify their contributions to forest structure and dynamics
Source: PLoS One. 2020 Dec 10;15(12):e0243079. doi: 10.1371/journal.pone.0243079 (PMC7728260; doi:10.1371/journal.pone.0243079)
Supplement: Attachment — Submitted filename: Response_to_reviewers.docx [file pone.0243079.s012.docx]

October 20, 2020

To:

Jana Müllerová, Academic Editor

PLOS ONE

With regards to our submission (PONE-D-20-08690R1), please find below the changes we have made to address the helpful comments of the reviewer. We have added the discussion of investigating the proportion of trees in the canopy per species/genus and addressed the minor suggestions. We thank the reviewers and editors for their contributions.

Sincerely,

Raquel Fernandes de Araujo

DETAILED RESPONSES IN ITALICS

**Reviewer #2**

Besides some details (see below), the only further suggestion I have relates to a comment of the previous review: I suggested to study the species as a factor of canopy proportions, DBH and growth rates. I undersand, that your data may not enable to do such analysis. Nevertheless, I would (briefly! - maybe 1-2 sentences) discuss that looking deeper into species/genus-specific relationships may shed additional light on the forest structural heterogeneity. Especially, considering that some species are by design shadow tolerant (there is no need to reach the canopy to thrive), whereas other species (competitive species) may only succeed on the long run, if the eventually reach the canopy. There may, for example, be only a few species with exceptional high canopy heights containing the majority of timber. I would consider to add such thoughts to 1) explain the scatter in your results and 2) provide an outlook for future work.

*R: As suggested, we added these thoughts in the second paragraph of discussion section. There the text now reads:*

*“Investigating the proportion of trees in the canopy per species or genus should bring more information on forest structure heterogeneity. At BCI, the proportion of gap-dependent species was higher in the canopy than the understory and increased with tree size, while the proportion of shade-tolerant species was smaller in the canopy and decreased with tree size [27]. Future studies should evaluate if the PPA can explain differences in the numbers and sizes of canopy trees among forests such as those between our site and BCI and explore differences in species and functional group compositions between canopy and understory.”*

Minor Details:

l.141 Remove full stop before bracket.

*R: Thank you for pointing this out. We removed the full stop before the bracket.*

l.149 Consider to state that you generated the products using Agisoft.

*R: We reformulate this statement. There the text now reads:*

*“We then generated a 3D point cloud, a digital elevation model (DEM) and a 2 cm spatial resolution orthomosaic (e.g., Fig 2, S4 File) using Agisoft Photoscan.”*

l.310 Full stop after and not before the bracket.

*R: We replaced the full stop after the bracket (this is in the l.242).*

Fig.5 In the text, you primarily refer to DBH, whereas in the figures you use ‘Diameter’ as axis label. Consider to also use DBH in the figures. Diameter is rather unspecific (and could, for example, also refer to crown diameter).

*R: Following the suggestion, we replaced the x axis labels of all figures with “DBH (cm)”.*
